# Electrostatic Potential of Functional Cations as a Predictor of Hydroxide Diffusion Pathways in Nanoconfined Environments of Anion Exchange Membranes

**DOI:** 10.1021/acs.jpclett.3c02800

**Published:** 2024-01-05

**Authors:** Tamar Zelovich, Dario R. Dekel, Mark E. Tuckerman

**Affiliations:** † Department of Chemistry, New York University (NYU), New York, New York 10003, United States; ‡ Wolfson Department of Chemical Engineering, Technion − Israel Institute of Technology, Haifa, 3200003, Israel; § Nancy & Stephen Grand Technion Energy Program, Technion − Israel Institute of Technology, Haifa, 3200003, Israel; ∥ Courant Institute of Mathematical Sciences, New York University (NYU), New York, New York 10012, United States; ⊥ NYU-ECNU Center for Computational Chemistry at NYU Shanghai, 3663 Zhongshan Rd. North, Shanghai 200062, China

## Abstract

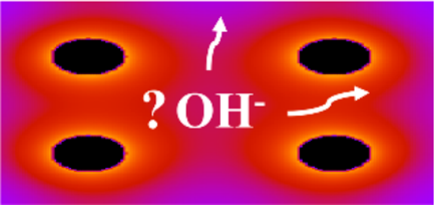

Nanoconfined anion exchange membranes (AEMs) play a vital role in emerging electrochemical technologies. The ability to control dominant hydroxide diffusion pathways is an important goal in the design of nanoconfined AEMs. Such control can shorten hydroxide transport pathways between electrodes, reduce transport resistance, and enhance device performance. In this work, we propose an electrostatic potential (ESP) approach to explore the effect of the polymer electrolyte cation spacing on hydroxide diffusion pathways from a molecular perspective. By exploring cation ESP energy surfaces and validating outcomes through prior *ab initio* molecular dynamics simulations of nanoconfined AEMs, we find that we can achieve control over preferred hydroxide diffusion pathways by adjusting the cation spacing. The results presented in this work provide a unique and straightforward approach to predict preferential hydroxide diffusion pathways, enabling efficient design of highly conductive nanoconfined AEM materials for electrochemical technologies.

Fuel cells and water electrolyzers employing anion exchange membranes (AEMs) are clean energy harvesting devices that produce clean energy and hydrogen, respectively, and are considered cost-effective^1−9^ due largely to the alkaline environment in which they operate, which eliminates the need for costly precious metal catalysts.^10−17^ Presently, the most challenging technical hurdle for AEM-based fuel cells and water electrolyzers is the development of membrane materials with high hydroxide ion conductivity and chemical stability.^12,13,18−22^

In recent years, nanoconfined environments have been exploited in the study of cost-effective and reliable polymer architectures,^23−35^ which are important components of emerging electrochemical device technologies.^36^ In functionalized nanoconfined structures of the type employed in the study of AEMs, one of the main goals is to gain control over the hydroxide ion diffusion pathways, as such a level of control can increase the hydroxide ion diffusion rate from the cathode to the anode, reduce the transport resistance, and generate highly conductive devices. Therefore, controlling the direction of the ion diffusion through a membrane may have a substantial impact on transport through ion-conducting materials and, in turn, on the performance of the aforementioned devices. However, a typical nanoconfined AEM is typically characterized as having random hydroxide diffusion directions across the membrane, which often result in an isotropic conductivity. Hence, the AEM community is currently facing a new challenge, namely, the exploration of ways to control the morphology of the diffusion channel and dominant pathways of the hydroxide ion diffusion in nanoconfined environments.^33,35,37−50^ For this purpose, the effect of morphology changes, membrane density, and electric or magnetic fields on ion diffusivity have been studied extensively.^37,41,46,48,51−53^ Some studies argued that morphological changes can affect ion diffusion pathways,^37,47^ while other studies suggested the use of electric or magnetic fields as a means of overcoming the random nature of hydroxide ion diffusion directions.^37,39,46,49,54^ For instance, Guiver and co-workers succeeded in constructing through-plane-oriented highly conductive hydroxide channels in ferrocenium AEMs using a magnetic field.^55^ In their study, AEMs designed for oriented ion transport, as compared with the isotropic (nonoriented) membrane reference, display substantially higher conductivity in the through-plane than the in-plane directions (ratio of through-plane conductivity/in-plane conductivity up to 36.8!). The through-plane-oriented morphology offers a route to further performance optimization in AEMs beyond conventional current approaches. Although this particular example of hydroxide transport orientation through AEMs was applied to fuel cells, the universal practicability of these materials may find additional applications in other areas of renewable and clean energy (e.g., electrolyzers) as well as in other areas that require oriented diffusion and mass transfer, including energy storage (e.g., battery separators and redox flow batteries), water technology (e.g., electrodialysis and reverse-osmosis membranes), and smart materials (e.g., artificial muscles).

The present work reports a theoretical investigation of the use of the electrostatic potential (ESP) energy profile of functional cations as a predictor of preferred diffusion pathways of hydroxide ions in model AEMs and an assessment of how knowledge of the ESP can serve as a tool to guide synthesis and experimental characterization in the design of polymer architectures that can generate distinctive hydroxide ion diffusion pathways. For this purpose, we calculate the ESP energy surface of the cations by using a lattice model as a mimic of the actual membrane structure. We validate the results using previously performed *ab initio* molecular dynamics (AIMD)^56,57^ simulations of three nanoconfined AEM models. The AIMD simulations are important to provide a molecular-level understanding of the predictability of ESPs on the diffusion pathways of hydroxide ions. Nonetheless, the findings indicate that future investigations can utilize the recommended ESP calculation to plot the ESP energy profile for any realistic polymer architectures in a nanoconfined setting, thus identifying preferred hydroxide-ion diffusion pathways without requiring computationally costly AIMD simulations.

We start by calculating the ESP energy profiles of an array of cations in the *xy* plane. The calculation does not include the water molecules or hydroxide ions that would normally exist in an AEM environment, yet we show that such inclusion is unnecessary. The model we propose comprises 100 cations, such that each cation is defined as a point in space carrying an electric charge of +1. The cations are located on a lattice, with 10 cations distributed along both the *x*- and *y*-directions, following the spacing specified by parameters of our previous AIMD studies.^58−66^ The model is further divided into a spatial grid, with a spacing of 0.1 Å in both the *x*- and *y*-directions. Finally, at each grid point, we calculate the contribution from all cations in the model according to eq 1
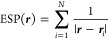
1where *N* in the number of cations in the system (i.e, *N* = 100), ***r*** is the two-dimensional vector (*x*, *y*), and ***r***_*i*_ = (*x*_*i*_, *y*_*i*_) is the coordinate vector of the *i*th cation. A spherical region of radius ∼1.5 Å around each cation is excluded from the calculation in eq 1. Equation 1 can also be generalized to a fully periodic lattice by including a sum over periodic images via
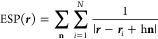
2where **n** is a two-dimensional vector of integers and h is the 2 × 2 cell matrix in the *xy* plane.

Using parameters from our previous AIMD simulations,^58−66^ we delineate two systems: system **A** and system **B**. The cation spacing in the *x*- and *y*-directions was set to Δ*x* = 10 Å for the two systems, while Δ*y* was chosen to be 8.7 Å for system **A** and 6.6 Å for system **B**. In Figure 1, we present the ESP energy profiles for systems **A** and **B**. To simplify the results presented in Figure 1, we plot the ESP of the four central cations. As shown, shorter cation distances correspond to higher ESP values in the regions between each pair of cations. Specifically, in system **B**, where cations are closer, the ESP energy profile demonstrates approximate uniformity throughout the cell. Furthermore, as the AIMD results will reveal, it becomes evident that the cation spacing in the *y*-direction for system **B** (i.e., 6.6 Å) is insufficient to permit the diffusion of hydroxide ions and water molecules. For system **A**, a heightened ESP value is observed between the cation pair along the *y*-direction, followed by a lower ESP value in the center-of-the-cell region (CCR) and between the cation pair along the *x*-direction. Our next step involves exploring how these findings can elucidate the observed preferred hydroxide diffusion pathways within AIMD simulations.

**Figure 1:**
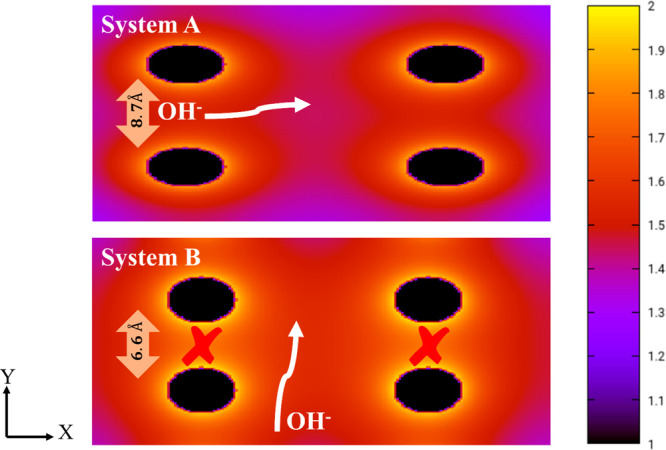
Electrostatic potential (ESP) energy profiles for the cations (excluding water molecules and hydroxide ions), along the *xy* plane for systems **b4** and **a4/a10** in their initial configuration, with the *z-*coordinate excluded from the calculation. The scale on the right provides the strength of the ESP.

As the primary objective of this study is to gain insight into how variations in cation spacing impact hydroxide-ion diffusion pathways through the estimation of cation ESP, we proceed to investigate three AEMs that were previously studied using AIMD simulations.^58−66^ For each model, we analyze the diffusion of the hydroxide ions and water molecules using methods described in our previous work.^58−66^ By leveraging the ESP energy profile presented in Figure 1, we aim to reveal how cation spacing influences hydroxide diffusion pathways.

For the AIMD simulations, each of the three nanoconfined AEM models contains two identical graphane layers aligned in the *xy*-plane, two trimethyl alkyl ammonium (TMA) cations attached to the graphane bilayers (GBs) using a (CH_2_)_2_ linker, two hydroxide ions (whose oxygen cores are denoted O*_1_ and O*_2_), and four or ten water molecules per cation (defined as the parameter λ). The two cations are attached via linkers to fixed points in the GBs but are otherwise free to move in aqueous solution. As indicated in Figure 2, the distance between the attachment points defines the polymer electrolyte cation spacing in the *x*- and *y*-directions. As a result, the simulation cell is partitioned into an open region in the center of the cell, which we refer to as CCR, and constricted regions between the cations, which we refer to as bottleneck regions (BRs). Using parameters from refs (67) and (68), we set the distance between the two carbon sheets, Δ*z*, at 7.3 and 7.8 Å for λ = 4 and 10, respectively (see the Supporting Information and refs (58−65) for the rationale). The polymer electrolyte cation spacing, measured between two nitrogen atoms in the *x*- and *y*-directions (Δ*x* and Δ*y*), was set to 10 Å for all systems in the *x* direction and 6.6 or 8.7 Å in the *y*-direction. In this way, the cation lattice model described above is an idealized representation of these three systems. The three systems are simulated at approximately room temperature. Once constructed, AIMD simulations^56,57^ were performed on the models using the CPMD code.^57,69^ A detailed description of the computational methods and the system parameters is presented in the Supporting Information and can be found in our previous work.^58−66^ For clarity, we refer to the three representative systems as **b4**, **a4**, and **a10** in which “a” and “b” represent the two Δ*y* values of 8.7 and 6.6 Å, respectively, and the numbers represent the hydration level. Furthermore, systems **a4** and **a10** replicate the cation spacing of system **A**, whereas system **b4** emulates the cation spacing of system **B**. It is worth mentioning that our model presents an idealized representation of the water morphology and ion diffusion paths. In our recent work, integrating theoretical and experimental approaches,^63,65^ we observed a notable agreement between the predicted hydroxide diffusion in our theoretical ideal model and corresponding experimental results. This alignment serves as compelling evidence, supporting the notion that despite its idealized nature, our model has the capacity to accurately predict hydroxide diffusion in realistic systems.

**Figure 2:**
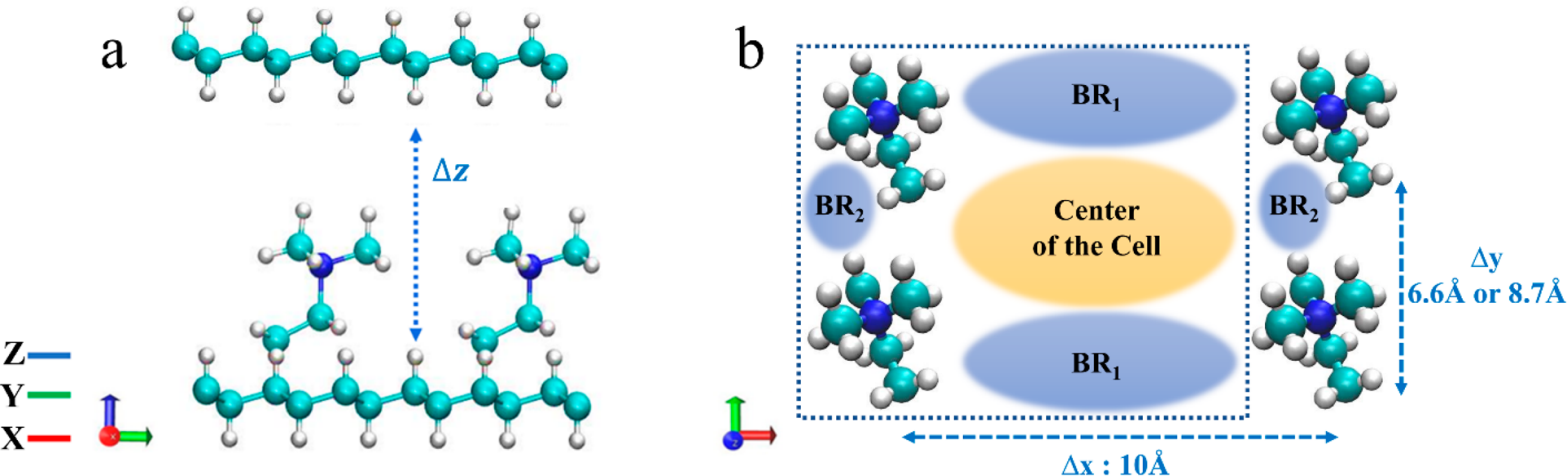
A typical cell (shown without water molecules), demonstrating the effective distance between the two graphane sheets along the *z*-axis (Δ*z*) on the left and the cation spacing along the *x-* and *y-*axes on the right (Δ*x* and Δ*y*). The blue and orange areas in the right figure indicate the bottleneck regions (BRs) and the center of the cell region (CCR), respectively. White, turquoise, and blue spheres represent H, C, and N atoms, respectively. The graphane bilayer atoms (C and H atoms) were removed from the right figure to better convey the cation structure. The light blue rectangle in the right panel shows the extent of the simulation cell.

In order to analyze the diffusion of both water and hydroxide ions in the AIMD simulations, we calculate the water and hydroxide diffusion constants separately along each spatial direction in the three systems (Table 1). As shown in Figure 2, the systems examined in this work have a unique cell shape that influences the motion of water molecules and hydroxide ions differently in each of three spatial directions. Therefore, in order to gain a deeper understanding of the hydroxide transport, we explore the motion and diffusion constants along each of the three axes separately. These components can be regarded as the diagonal elements of the diffusion tensor, an important quantity in the calculation of ionic conductivities.^58−66^

**Table 1:** Diffusion Coefficients Obtained from the Slope of the Mean Square Displacement (MSD) in Units of 1 × 10^–8^ m^2^/s (i.e., Å^2^/ps)

	***D***_**OH^*–*^**_				***D***_**H_2_O**_			
**System**	***D***	***D*_*X*_**	***D*_*Y*_**	***D*_*Z*_**	***D***	***D*_*X*_**	***D*_*Y*_**	***D*_*Z*_**
**b4**	0.17	0.06	0.42	0.03	0.06	0.04	0.12	0.02
**a4**	0.13	0.34	0.04	0.02	0.25	0.52	0.20	0.03
**a10**	0.20	0.52	0.03	0.06	0.08	0.20	0.03	0.01
**Bulk Solution**	0.45^a^				0.17^b^			

a Results taken from ref (70) using the B-LYP functional.

b Results taken from ref (71) using the B-LYP functional. See the Supporting Information for MSD curves.

In order to provide additional insight into the diffusion coefficients presented in Table 1, we plot the coordinates of the hydroxide ion oxygens in each system as a function of time along the *x-* and *y-*axes separately in Figure 3 (in the Supporting Information, we label proton transfer (PT) events on top of the hydroxide ion coordinates).

**Figure 3:**
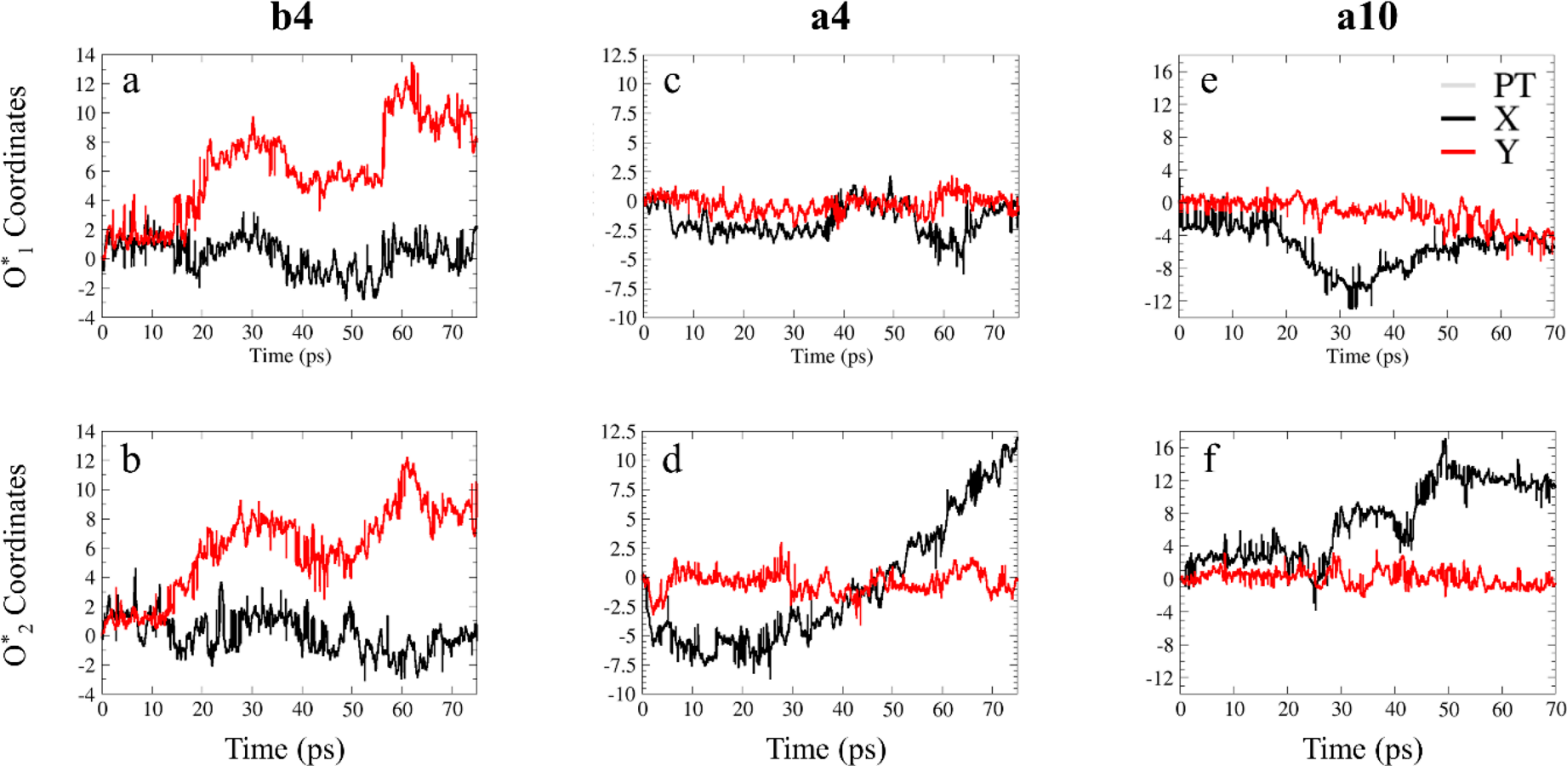
Hydroxide ion oxygen coordinates as a function of time (black and red curves for *x* and *y* coordinates, respectively) for O*_1_ and O*_2_ during the simulations for systems **b4**, **a4**, and **a10**. The hydroxide ion coordinates along the *z-*axis are not presented, as they contribute negligibly to the overall diffusion.

In order to explore the preferential hydroxide-ion diffusion pathways in the AIMD simulations, in Figures 4A and C, we present the spatial populations in the *xy-*plane for each of the two hydroxide ions as generated from the NVE trajectory of systems **b4** and **a4**. Parts B and D of Figure 4 present a representative configuration demonstrating the hydroxide ion diffusion extracted directly from the AIMD trajectories. This allows us to learn the preferred locations of the hydroxide ions with respect to the cations and provides a clear picture of the hydroxide diffusion pathways along the *x-* and *y-*axes.

**Figure 4:**
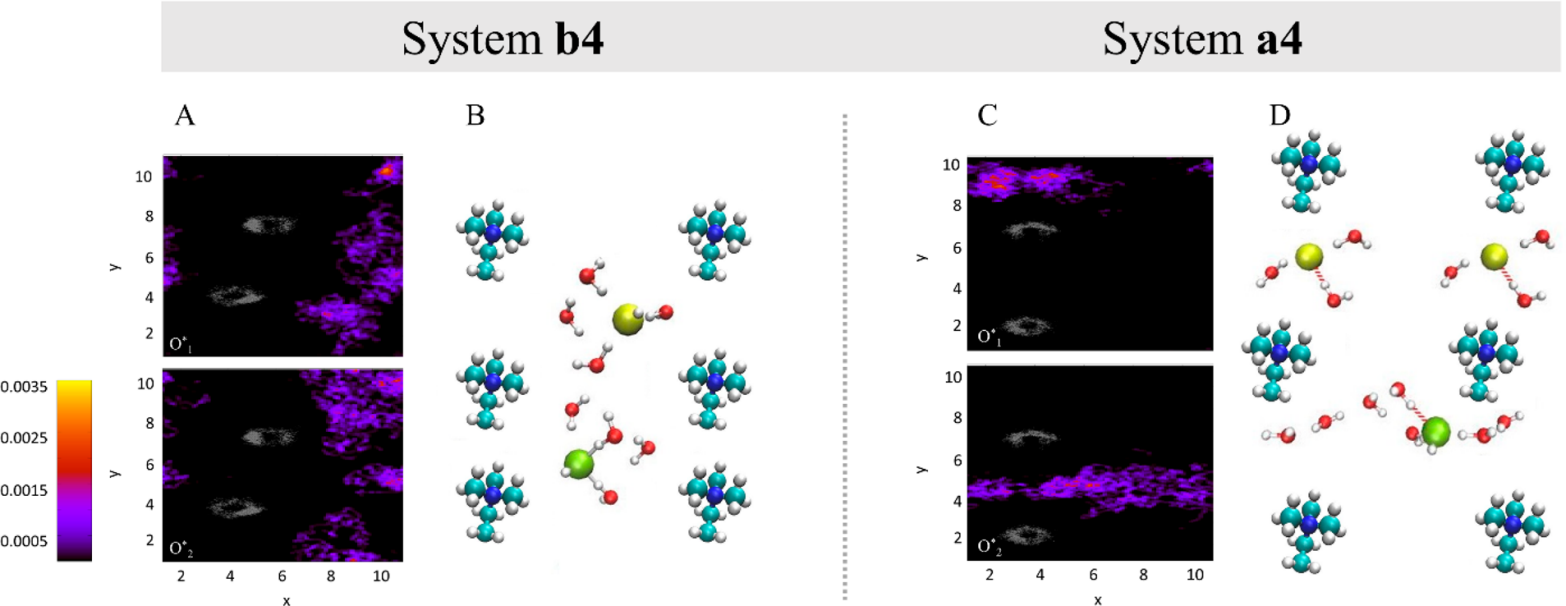
(A, C) Spatial population of the two hydroxides (upper and lower panels for O*_1_ and O*_2_) of system **b4** at 295 K and system **a4** at 300 K. The gray areas represent the locations of the cations throughout the simulations, and the color bar represents the probability density of the location of hydroxide ions in the *xy* plane, independent of their *z* coordinates, normalized by the number of steps from the NVE trajectory. (B, D) Representative configurations showing the hydroxide diffusion mechanism for system **b4** at 295 K and system **a4** at 300 K, including significant water molecules from the first and second solvation shells. Red, white, turquoise, and blue spheres represent the O, H, C, and N atoms, respectively, and yellow spheres represent the current hydroxide.

In our previous AIMD studies on AEM mimics,^58−66^ we found that, in low-hydrated lamellar-like GB structures, the narrow area between each pair of TMA cations is a bottleneck that suppresses hydroxide-ion diffusion. This allows only certain solvation structures to be mobile through these BRs.^58,59,61,63^ The BRs are determined by the polymer electrolyte cation spacing in the *x*- and *y*-directions. For the purpose of this study, we define two BRs (Figure 2): BR_1_ is defined by Δ*x*, and BR_2_ is defined by Δ*y*. While BR_1_ is identical for all systems and is equal to 10 Å, BR_2_ is around 6.6 Å for system **b4** and 8.7 Å for systems **a4** and **a10**. We find that BR_2_ in system **b4** is too narrow to allow diffusion of hydroxide ions and water molecules; therefore, all species diffuse only in the *y*-direction. This agrees with the results presented in Table 1, as the diffusion coefficients of the hydroxide ions in the *x*- and *y*-directions were found to be 0.06 and 0.42 × 10^–8^ m^2^/s, respectively, and the diffusion coefficients of the water molecules in the *x*- and *y*-directions were found to be 0.04 and 0.12 × 10^–8^ m^2^/s, respectively (the differences between the water and hydroxide ion diffusion coefficients were discussed in our previous work^58,59^). Moreover, the diffusion of the hydroxide ions in the *y-*direction agrees with the hydroxide-ion coordinates presented in Figure 3, where we find significant diffusion along the *y-*axis for both hydroxide ions with no diffusion along the *x-*axis. The spatial populations of the two hydroxide ions in system **b4** (Figure 4A) support the picture of hydroxide diffusion in the *y*-direction along BR_1_ while also supporting the claim that BR_2_ is too narrow for hydroxide diffusion. Figure 4B presents a representative configuration for the hydroxide solvation structure during diffusion along the *y-*axis. Comparison of the NO* radial distribution functions (RDFs) of the three systems presented in Figure 5 shows a narrower peak for system **b4**. This suggests that the hydroxide ions in system **b4** diffuse in the CCR throughout most of the simulation at a constant distance from the TMA cations. This observation is in agreement with the ESP surface presented in Figure 1, which exhibits a constant and uniformly distributed ESP along the diffusion path in the *y*-direction. A full analysis of the hydroxide ion solvation structure and diffusion mechanism in system **b4** can be found in our previous work.^58,59,63^

**Figure 5:**
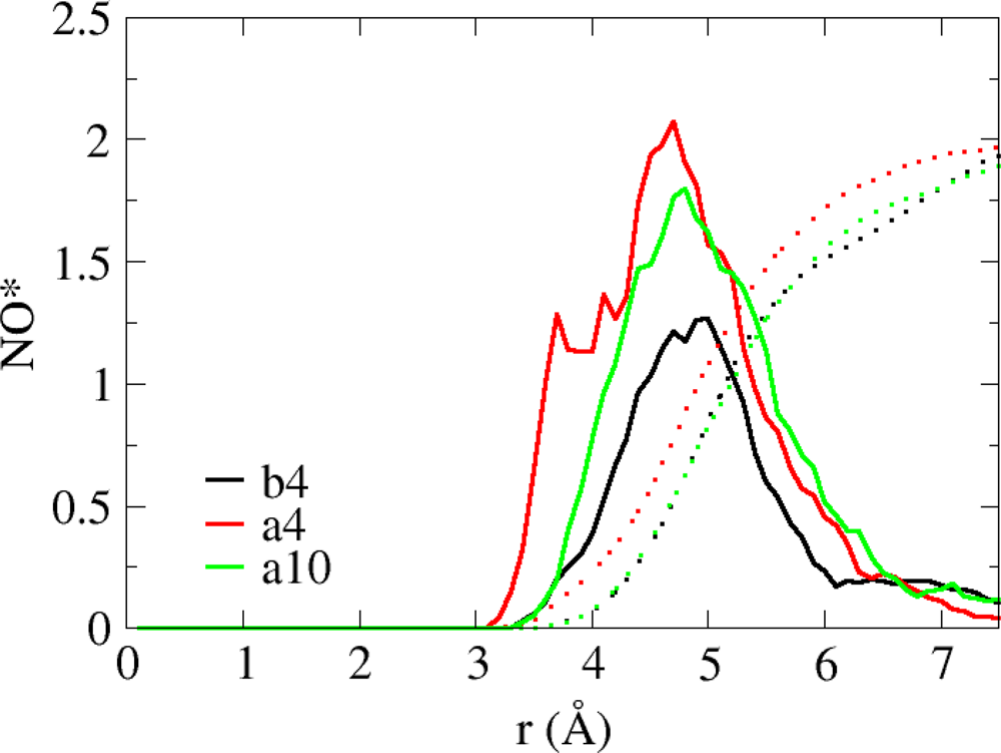
NO* radial distribution functions (RDFs) for systems **b4**, **a4**, and **a20** are shown in black, red, and green curves, respectively. The dashed lines represent the running coordination numbers (CNs).

Systems **a4** and **a10** present more complex hydroxide diffusion as these systems contain two BRs that allow for hydroxide and water diffusion. As mentioned, only certain solvation structures can diffuse through these BRs.^58,59,61,63^ The wider the BR, the easier it will be for the hydroxide ions to achieve these structures; hence, we would expect to find a higher hydroxide diffusion rate in the wider BRs (i.e., along the *y*-axis). However, as suggested by the diffusion coefficients presented in Table 1, the hydroxide ions in systems **a4** and **a10** diffuse primarily along the narrower BR (i.e., along the *x*-axis), with hydroxide diffusion constants in the *x*-direction of 0.34 and 0.52 × 10^–8^ m^2^/s and 0.04 and 0.03 × 10^–8^ m^2^/s in the *y-*direction, for systems **a4** and **a10**, respectively. To support these results, we use the hydroxide ion coordinates presented in Figure 3, where we find that, for system **a4**, O*_1_ is nondiffusive while O*_2_ undergoes mainly vehicular diffusion in the *x*-axis (i.e., the narrower BR), and for system **a10**, we observe structural diffusion in the *x*-axis for both hydroxide ions (i.e., the narrower BR). These counterintuitive results suggest that there is a clear preference for diffusion along the narrower channel. The tendency of the hydroxide ion pathways toward the narrower diffusion pathways can be explained by a stronger coulomb interaction between the hydroxide ions and the TMA cations in the narrower channel (i.e., the *x*-direction), as seen in the ESP presented in Figure 1. Specifically, we find a reduction in the ESP within the CCR and an elevation of ESP in the region between each pair of TMA cations, with a more pronounced ESP increase between the pair of cations along the *y*-direction compared to the *x*-direction. The NO* RDFs presented in Figure 5 display wider peaks for systems **a4** and **a10** compared to system **b4**, suggesting that the hydroxide ions in systems **a4** and **a10** are located closer to the cations as a result of the stronger coulomb interaction, yet they are located further away from the cations when diffusing along the CCR. To further support the observation that hydroxide ions exhibit a preference for diffusion within the narrower BR, we refer to the spatial population probability presented in Figure 4C. This graphical representation reveals that the hydroxide ions within system **a4** tend to be situated more frequently near the TMA cations or along the BRs with a comparatively lower likelihood of being found in the CCR. Figure 4D presents a representative configuration for the hydroxide ion solvation structure during diffusion along the *y-*axis. A full analysis of the hydroxide diffusion mechanism in systems **a4** and **a10** can be found in our previous work.^58−60,63^ In the Supporting Information, we present the water density profile, OO and O*O RDFs, and CNs of the three systems to support the results presented in this study.

It should be noted that the three simulations reported in this study were carried out in the temperature range of 290–330 K. In our recent study exploring the temperature effect on hydroxide diffusion,^63^ we find that, at higher temperatures, the hydroxides can escape the hydroxide/cation coulomb attraction, allowing the hydroxide ions to diffuse in any direction allowed by the arrangement of the cations.

AIMD simulations are considered an appropriate and accurate approach for exploring ion dynamics as the interatomic forces are generated from electronic structure calculations performed “on the fly” as the simulation proceeds. However, AIMD simulations carry a significant computational overhead. Combining AIMD simulations with the ESP model presented in eq 1 has provided us with intriguing yet counterintuitive results concerning preferred hydroxide diffusion pathways and the influence of the cation spacing in nanoconfined structures. These findings could have important implications for materials design. An ability to control the relative diffusion of ions along particular directions may give substantial advantages in the design of membranes for electrochemical devices, where through-plane diffusion pathways are required. The use of AIMD simulations was important in this study in order to provide a molecular level picture and validate the predictions of the ESP model on the hydroxide-ion diffusion pathways. However, going forward, based on the results provided in this study, simply plotting the ESP for any choice of cation spacing in any nanoconfined environment, and for any realistic polymer architectures, using the suggested ESP approach can reveal the preferred hydroxide-ion diffusion pathways without requiring computationally costly AIMD calculations.

In summary, we investigated the predictability of an electrostatic potential (ESP) model of preferred hydroxide diffusion pathways and validated those predictions using previously performed AIMD simulations. Controlling hydroxide diffusion pathways in nanoconfined environments is desirable in the design of highly conductive and efficient AEMs, as this control can shorten the hydroxide diffusion pathways between the electrodes, reduce the transport resistance, and improve the AEM performance. Using three GB models and corresponding idealized lattice models for computing the ESP, we demonstrated how by changing a single parameter, such as the cation spacing, we can create distinctive hydroxide ion diffusion pathways and predict which pathway is more favorable for hydroxide diffusion, especially in scenarios with multiple pathways. Although it is generally easier for requisite hydroxide solvation structures to form in wider BR regions, our results reveal an unexpected preference for hydroxide ions to diffuse primarily along narrower BRs. This counterintuitive behavior is attributed to a competition between wider BRs allowing easier solvation versus narrower channels exhibiting a stronger coulomb interaction between the hydroxide ions and the TMA cations. These nontrivial findings underscore the intricate interplay between channel width and electrostatic forces in governing hydroxide ion diffusion.

The results presented herein allow us to provide a unique design model and a powerful predictive tool that can guide synthesis and experimental characterization in the engineering of membranes, in which the preferred hydroxide diffusion pathways can be tailored and easily controlled. This will lead to the development of new, highly efficient, and advanced AEM materials. Although this particular example, which demonstrates the identification of preferred hydroxide diffusion pathways in model AEMs, was presented in the context of fuel cells and water electrolyzers, the universal practicability of these materials may find additional applications in other areas, including other ionomeric materials. The possibility of controlling dominant ion diffusion pathways in ion-conducting membranes could potentially pave the way toward the design of new materials, leading to high-performing electrochemical devices and applications.

## Supporting Information Available

The following additional data are presented for all systems: (i) computational methods and system parameters; (ii) water structure; (iii) OO and O*O RDFs and CNs; (iv) hydroxide ion coordinates as a function of time; (v) MSD plots; and (vi) log–log MDS plots (PDF)
