# Supplementary material for: Electrostatic Potential of Functional Cations as a Predictor of Hydroxide Diffusion Pathways in Nanoconfined Environments of Anion Exchange Membranes
Source: J Phys Chem Lett. 2024 Jan 5;15(2):408–15. doi: 10.1021/acs.jpclett.3c02800 (PMC10801687; doi:10.1021/acs.jpclett.3c02800)
Supplement: Supplementary file 1 — jz3c02800_si_001.pdf [file jz3c02800_si_001.pdf]

Electrostatic Potential of Functional Cations as A Predictor of Hydroxide Diffusion Pathways in Nanoconfined Environments of Anion Exchange Membranes

Supporting Information

Tamar Zelovich[(1)] , Dario. R. Dekel[(2),(3)], *Mark E. Tuckerman[(1),(4),(5)]

1) Department of Chemistry, New York University (NYU), New York, New York 10003, United States
2) Wolfson Department of Chemical Engineering, Technion – Israel Institute of Technology, Haifa, 3200003, Israel
3) Nancy & Stephen Grand Technion Energy Program, Technion – Israel Institute of Technology, Haifa, 3200003, Israel
4) Courant Institute of Mathematical Sciences, New York University (NYU), New York, New York 10012, United States
5) NYU-ECNU Center for Computational Chemistry at NYU Shanghai, 3663 Zhongshan Rd. North, Shanghai 200062, China

Computational Methods

Each AEM system is constructed using a set of geometric requirements. We start by choosing a shape for the graphane bilayer (GB) setup that allows for periodic replication of the confined structure, in this case the cell lengths in the periodic directions *x* and *y*. We then choose the composition of the linker as $(CH_2)_2$ and attach two tetramethylammonium (TMA) to one side of the GB. Next, we add a selected number of TIP3P water molecules [(1)], previously equilibrated in a classical MD bulk water simulation (using the TIP3P potential) [(1)], and hydroxide ions between the graphane sheets by overlaying the bulk water simulation with the confined structure and selecting molecules with no spatial overlap with the cations. Finally, we set the distance between the two graphane layers, Δz, calculated as the distance between the hydrogen atoms on the inner surfaces. All water molecules fit in the spaces between the cations, and hence, Δz is set to the maximum possible height of the cations, which is 7.3 Å and 7.8 Å for λ = 4 and 10, respectively.

After construction of the initial structures, *ab initio* molecular dynamics (AIMD) simulations [(2,3)] were run using the CPMD code. [(4)] We employed the dispersion-corrected atomic core pseudopotentials (DCACP) scheme[(5)] within the Kohn-Sham formulation of density

functional theory (DFT) in order to ensure adequate treatment of the dispersion forces, and the B-LYP exchange-correlation functional [(6,7)], which has proven to be accurate in the treatment of the aqueous hydroxide ion.[(8)] A plane-wave (PW) basis set was employed to expand the Kohn-Sham orbitals with an energy cutoff of 80 Ry. The simulations were carried out using the mass of D instead of H for all hydrogen atoms in order to allow a larger time step to be employed and to reduce the importance of nuclear quantum effects.[(9)] The fictitious mass of the expansion coefficients was taken to be μ=600 a.u., and a time step of 4 a.u. (0.096 fs) was employed for all simulations. Each system was equilibrated at the desired temperature using a massive Nosé-Hoover chain thermostat [(10)], followed by 15-20 ps of canonical (NVT) dynamics, also using a Nosé-Hoover chain thermostat, finally followed by ~80 ps of microcanonical (NVE) dynamics. The initial temperature for each NVE simulation is the final temperature obtained in the NVT simulation (which necessarily fluctuates around the target temperature). The temperature of the NVE simulation then oscillates around this initial temperature, as the temperature is not controlled under NVE. Due to deviations from the initial temperature set during the NVT run, the average temperature over the NVE trajectory is reported, rounded to the nearest five Kelvin.

For each atomic configuration generated in a simulation, the hydroxide ions in the system, whose oxygen atoms are designated as O*, were identified by finding the two oxygen atoms with only a single covalent hydrogen bond. Since each hydrogen can be uniquely assigned to a single oxygen based on the minimum O-H bond length, this assignment is unambiguous. All radial distribution functions were calculated using both the NVT and NVE trajectories, while all dynamic properties were obtained using only the NVE trajectories.

Systems Parameters

Table S1: System parameters for the three graphane bilayer (GBs) structures presented in this study.

System	T (K)	Hydration Level (λ)	Cation Spacing (Å)		Cell Geometry (Å)		
			x-axis	y-axis	x-axis	y-axis	z-axis
**b4**	295	4	10.064	6.6	10.064	13.07	7.3
**a4**	300	4	10.064	8.7	10.064	17.43	7.3
**a10**	330	10	10.064	8.7	10.064	17.43	7.8

Water Structure

We begin characterizing the water structure under nano-confinement by plotting the oxygen atom distribution along the *z*-axis for each of the GB systems (see Figure S1). For system 10, we find two peaks, which indicates for the existence of water layers with water depletion between them. The existence of two similar peak heights, at 3.7 Å and 6.4 Å, suggests two similar water layers, with a distance of ~2.7 Å between them. A more detailed discussion of the water structure in system **a10** can be found in our previous work, Refs. (11), where we referred to system **a10**, as **GB10a**.

For systems **b4** and **a4**, the distance between the two carbon sheets is too narrow to create two water layers. According to the O_wO_w radial distribution functions (RDFs) and coordination numbers (CNs) presented in Figure S2a, the first solvation shell of water oxygens is comprised, on average, of ~1.5 oxygens. These results, together with inspection of configurations from the NVE trajectory, reveal that the water structure alternates between a uniform wire structure, in which the water oxygens are in a twofold structure, and a non-uniform water distribution, in which the water molecules are separated into groups of three or four water molecules coordinating each hydroxide ion, with an average distance larger than 4Å between two groups of associated waters. This clustering results in a first solvation shell of either zero or one for the water oxygens. [11]

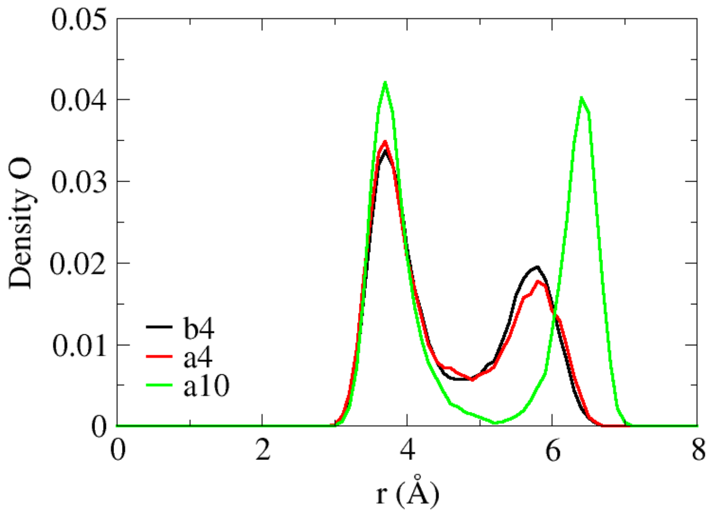

Figure S1: Oxygen atom distribution along the *z*-axis for systems **b4**, **a4**, and **a10** (black, red, and green curves, respectively).

OO and O*O Radial Distribution Functions

In our previous study of AEMs under low hydration conditions ($2 < \lambda < 5$), we found that a system should be considered to lie within the low-hydration regime if the water is distributed nonuniformly throughout the AEM. Factors such as hydration level, cation spacing, and the distance between the two GB, were all found to affect the water distribution throughout the cell. As discussed in the previous section, O_wO_w RDFs and CNs (Figure S2a) confirms the non-uniform water distribution for systems **b4** and **a4**, with CN values lower than 2 for the first solvation shell.

See our previous work, Refs. (11-15) for a full analysis of the hydroxide ions and water structure and diffusion mechanisms in systems **b4**, **a4**, and **a10**.

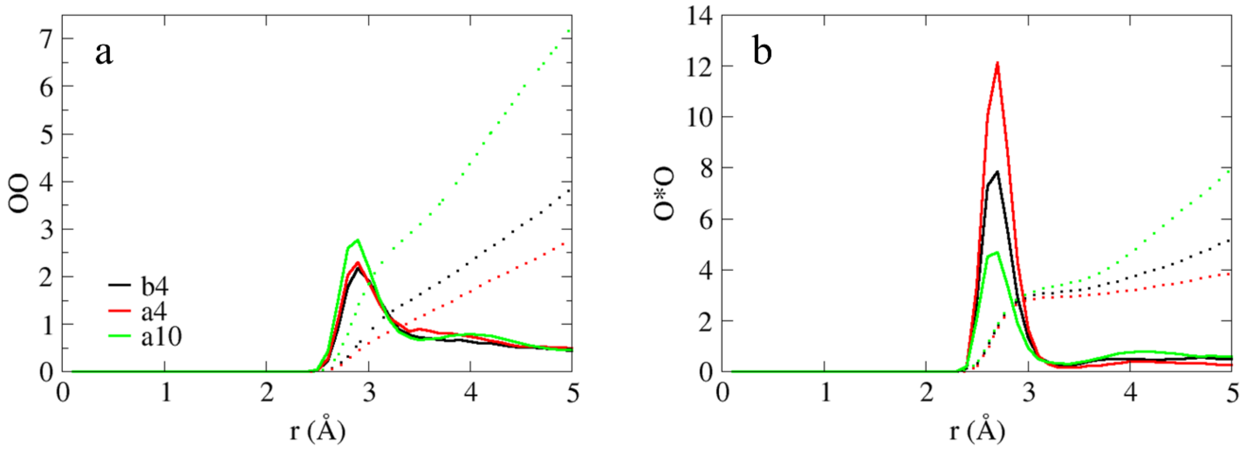

Figure S2: (a) OO and (b) O*O radial distribution functions (RDFs) for systems **b4**, **a4**, and **a20** in black, red, and green curves, respectively. The dashed lines represent the running coordination numbers (CNs).

Hydroxide Ion Oxygen Coordinates

Figure S3, presents the hydroxide ion coordinates as a function of time (as presneted in Figure 3 of the main text), and label proton transfer (PT) events (excluding events in which the proton transfers forth and back before transferring to a third oxygen, known as "rattling" events) with gray lines, where each line represents a change in the identity of the hydroxide oxygen. A smooth change in the hydroxide ion oxygen coordinates is indicative of vehicular diffusion [(11)], while a sharp jump in the coordinates of one of the OH^- oxygens, caused by a PT event, is associated with structural diffusion [(8,16-20)], often referred to as the "Grotthuss diffusion". Using

this information, we analyzed the hydroxide ions diffusion mechanisms in our previous work, Refs. (11-15) .

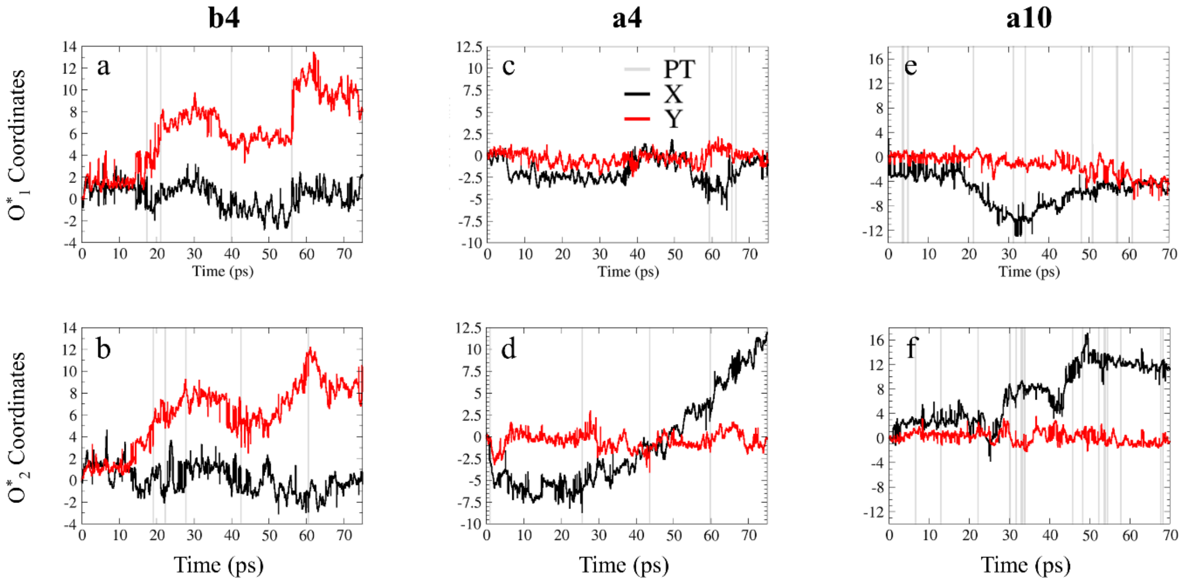

Figure S3: Hydroxide ion oxygen coordinates as a function of time (black and red curves for *x* and *y* coordinates, respectively) for O^*_1 and for O^*_2 during the simulations for systems **b4**, **a4** and **a10**. Gray lines indicate PT events (excluding rattling) that result in a change of the hydroxide ion identity.

Mean Square Displacements

Figure S4 presents the mean square displacement (MSD) curves as functions of time, calculated for each of the three spatial directions separately and as an average over the three directions, for the three systems in the main text. The results were used for the calculation of the diffusion constants presented in the main text. In all systems, the results presented were obtained from the first 10% of the NVE trajectory in order to show the transition to the diffusive-linear behavior appears approximately after 2ps. However, for the diffusion calculation, the data used was obtained from the first 2% to the first 10% of the NVE trajectory.

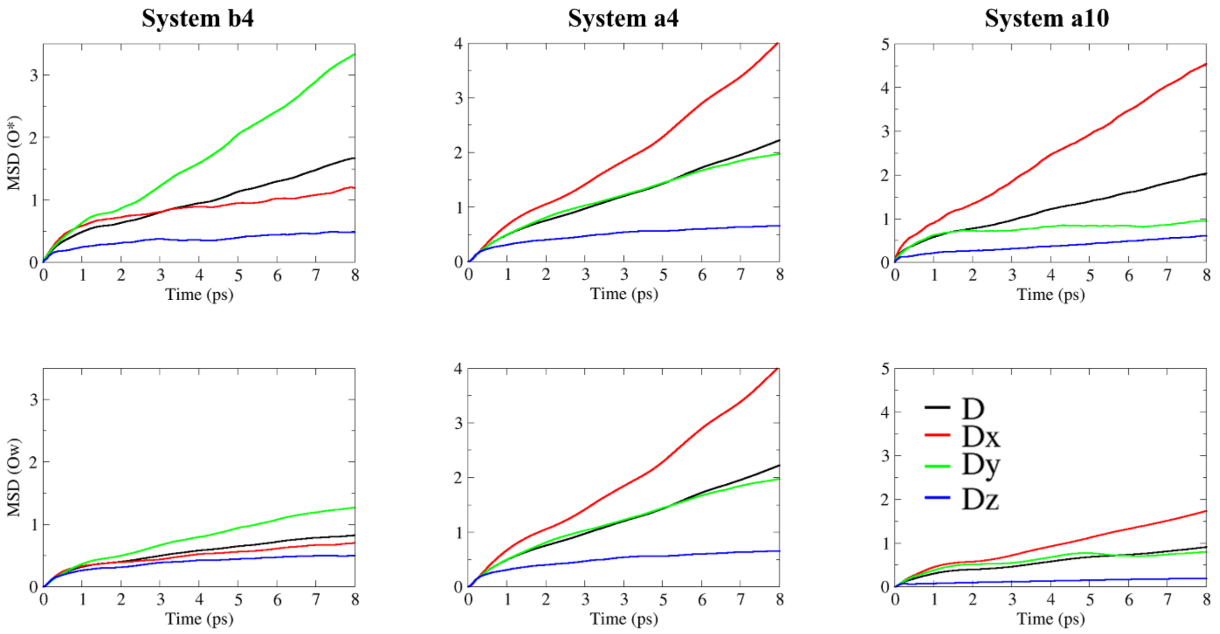

Figure S4: Mean square displacement (MSD) for OH^- (upper panels) and H_2O (lower panels) as a function of time, calculated as an average (black curve) and for each of the axes separately (red, green and blue represents *x*-, *y*- and *z*- axes, respectively) for the three systems.

<u>Figure S5: log-log MSD vs Time</u>

In bulk solutions, the MSD log-log plot [(21)] is characterized by three distinct regions of behavior. The initial region, termed 'free motion,' occurs at very short observation times, exhibiting a slope of less than 0.5. The intermediate region, at moderate times, features a slope between 0.5 and 1. The third and final regime is the long-time behavior where the slope of the MSD log-log plot is 1. For bulk solution studies, simulations must reach the third regime in order for an accurate diffusion coefficient to be determined. However, in confined geometries, where single file diffusion occurs, the slope of the MSD log-log plot in the long-time regime was also found to be between 0.5 to 1. [(22-25)] This diffusion behavior is known as a sub-diffusion regime.[(22)]

To demonstrate the accuracy of our diffusion coefficients calculations in the context of confined systems, we present in Figure S5 the log-log MSD as a function of time for systems **a4** and **b4** – the two main systems discussed in the main text. As shown, the slopes of the MSD log-log plots align with the sub-diffusion regime, as the values are between 0.5 to 1. For each

system, the average diffusion coefficient for OH^{-} and H_2O are presented with black and red lines, respectively.

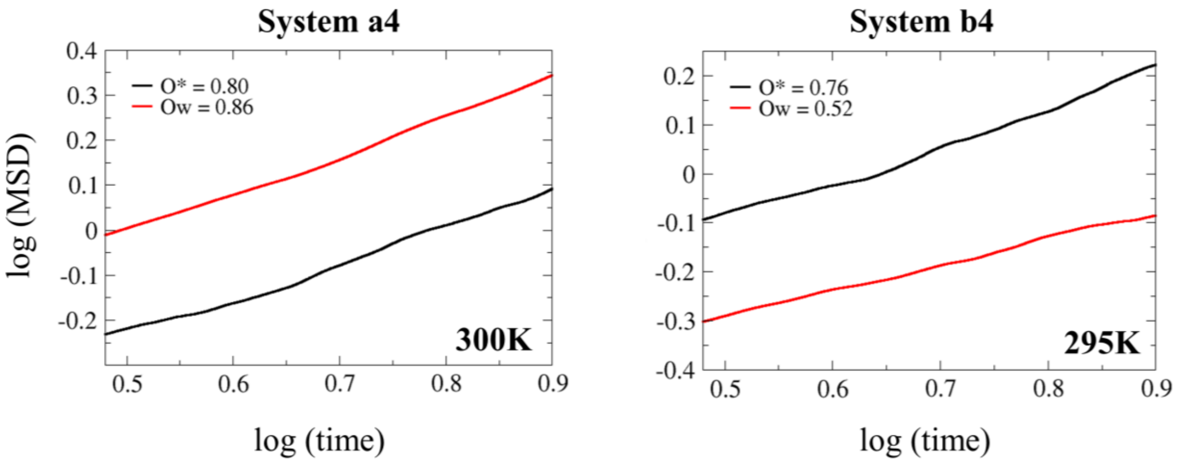

Figure S5: MSD log-log plot for OH^{-} (black curve) and H_2O (red curves) as a function of time, for systems **a4** and **b4**.

References

(1) P. Mark and L. Nilsson, “Structure and Dynamics of the TIP3P, SPC, and SPC/E Water Models at 298 K,” *J. Phys. Chem. A*, **2001**, *105*, 9954–9960.

(2) M. E. Tuckerman, “Ab Initio Molecular Dynamics: Basic Concepts, Current Trends and Novel Applications,” *J. Phys. Condens. Matter*, **2002**, *14*, R1297–R1355.

(3) D. Marx and J. Hutter, “*Ab Initio Molecular Dynamics: Theory and Implementation, in Modern Methods and Algorithms of Quantum Chemistry”*, Vol.1. Juelich: Forschungszentrum, 2000.

(4) D. M. J. Hutter, A. Alavi, T. Deutsch, M. Bernasconi, S. Goedecker and M. T. and M. Parrinello, “CPMD, IBM Corporation 1990–2009 and MPI für Festkörperforschung 1997–2001; see www.cpmd.org, 2009.” .

(5) I.-C. Lin, M. D. Coutinho-Neto, C. Felsenheimer, O. A. von Lilienfeld, I. Tavernelli, and U. Rothlisberger, “Library of Dispersion-Corrected Atom-Centered Potentials for Generalized Gradient Approximation Functionals: Elements H, C, N, O, He, Ne, Ar, and

Kr," *Phys. Rev. B,* **2007**, *75,* 205131–205135.

(6) A. D. Becke, "Density-Functional Exchange-Energy Approximation With Correct Asymptotic Behavior," *Phys. Rev. A,* **1988**, *38,* 3098–3100.

(7) C. Lee, W. Yang, and R. G. Parr, "Development of the Colle-Salvetti Correlation-Energy Formula into A Functional of the Electron Density," *Phys. Rev. B,* **1988**, *37,* 785–789.

(8) M. E. Tuckerman, A. Chandra, and D. Marx, "Structure and Dynamics of OH^- (aq)," *Acc. Chem. Res.,* **2006**, *39,* 151–158.

(9) D. Marx and J. Hutter, "*Ab Initio Molecular Dynamics: Basic Theory and Advanced Methods"*, Cambridge. Cambridge: Cambridge University Press, 2009.

(10) G. J. Martyna and M. L. Klein, "Nose-Hoover Chains: The Canonical Ensemble via Continuous Dynamics," *J. Chem. Phys.,* **1992**, *97,* 2635–2643.

(11) T. Zelovich, Z. Long, M. Hickner, S. J. Paddison, C. Bae, and M. E. Tuckerman, "Ab *Initio* Molecular Dynamics Study of Hydroxide Diffusion Mechanisms in Nano-Confined Structural mimics of Anion Exchange Membranes," *J. Phys. Chem. C,* **2019**, *123,* 4638–4653.

(12) T. Zelovich, L. Vogt-Maranto, M. A. Hickner, S. J. Paddison, C. Bae, D. R. Dekel, and M. E. Tuckerman, "Hydroxide Ion Diffusion in Anion Exchange Membranes at Low Hydration: Insights from Ab *Initio* Molecular Dynamics," *Chem. Mater,* **2019**, *31,* 5778–5787.

(13) T. Zelovich and M. E. Tuckerman, "Water Layering Affects Hydroxide Diffusion in Functionalized Nanoconfined Environments," *J. Phys. Chem. Lett,* **2020**, *11,* 5087–5091.

(14) T. Zelovich and M. E. Tuckerman, "OH^- and H_3O^+ Diffusion in Model AEMs and PEMs at Low Hydration: Insights from Ab *Initio* Molecular Dynamics," *Membranes (Basel).,* **2021**, *11,* 355–368.

(15) T. Zelovich, L. Voth-Maranto, Cataldo Simari, Isabella Nicotera, M. A. Hickner, C. Bae, S. J. Paddison, Dario R. Dekel, and M. E. Tuckerman, "Non-Monotonic Temperature Dependence of Hydroxide Diffusion in Anion Exchange Membranes," *Chem. Mater.,*

2022, *23*, 2133-2145.

(16) M. E. Tuckerman, D. Marx, and M. Parrinello, "The Nature and Transport Mechanism of Hydrated Hydroxide Ions in Aqueous Solution," *Nature*, **2002**, *417*, 925–929.

(17) A. Chandra, M. E. Tuckerman, and D. Marx, "Connecting Solvation Shell Structure to Proton Transport Kinetics in Hydrogen – Bonded Networks via Population Correlation Functions," *Phys. Rev. Lett*, **2007**, *99*, 145901–145904.

(18) M. E. Tuckerman, A. Chandra, and D. Marx, "A Statistical Mechanical Theory of Proton Transport Kinetics in Hydrogen-Bonded Networks Based On Population Correlation Functions With Applications to Acids and Bases," *J. Chem. Phys.*, **2010**, *133*, 124108–124129.

(19) Z. Ma and M. E. Tuckerman, "On the Connection Between Proton Transport , Structural Diffusion, and Reorientation of the Hydrated Hydroxide Ion as a Function of Temperature," *Chem. Phys. Lett*, **2011**, *511*, 177–182.

(20) D. Marx, A. Chandra, and M. E. Tuckerman, "Aqueous Basic Solutions : Hydroxide Solvation , Structural Diffusion , and Comparison to the Hydrated Proton," *Chem. Rev.*, **2010**, *110*, 2174–2216.

(21) E. J. Maginn, R. A. Messerly, D. J. Carlson, D. R. Roe, and E. J. R., "Best Practices for Computing Transport Properties 1. Self-Diffusivity and Viscosity from Equilibrium Molecular Dynamics," *Living J. Comp. Mol. Sci*, **2019**, *1*, 6324–6344.

(22) U. Siems, C. Kreuter, A. Erbe, N. Schwierz, S. Sengupta, P. Leiderer, and P. Nielaba "Non-Monotonic Crossover from Single-File to Regular Diffusion in Micro-Channels," *Sci. Rep.*, **2012**, *2*, 1015–1019.

(23) M. Zhao and X. Yang, "Segregation Structures and Miscellaneous Diffusions for Ethanol/ Water Mixtures in Graphene-Based Nanoscale Pores," *J. Phys. Chem. C*, **2015**, *119*, 21664−21673.

(24) U. Siems and P. Nielaba, "Single-File Diffusion in A Multilayered Channel," *Phys. Rev. E*, **2018**, *98*, 032127–032133.

(25) S. Ahmadi, M. Schmidt, R. J. Spiteri, and R. K. Bowles, "The Effect of Soft Repulsive Interactions on the Diffusion of Particles in Quasi-One Dimensional Channels: A Hopping Time approach," *J. Chem. Phys.*, **2019**, *150*, 224501–224511.
